# Elucidating the dual role of grain boundaries as dislocation sources and obstacles and its impact on toughness and brittle-to-ductile transition

**DOI:** 10.1038/s41598-020-59405-5

**Published:** 2020-02-17

**Authors:** Jens Reiser, Alexander Hartmaier

**Affiliations:** 1 Karlsruhe Institute of Technology, Institute for Applied Materials, 76344 Eggenstein-Leopoldshafen, Germany; 2 Ruhr-Universität Bochum, Interdisciplinary Centre for Advanced Materials Simulation, 44780 Bochum, Germany

**Keywords:** Mechanical properties, Metals and alloys

## Abstract

In this paper, we resolve the role of grain boundaries on toughness and the brittle-to-ductile transition. On the one hand, grain boundaries are obstacles for dislocation glide. On the other hand, the intersection points of grain boundaries with the crack front are assumed to be preferred dislocation nucleation sites. Here, we will show that the single contributions of grain boundaries (obstacles vs. source) on toughness and the brittle-to-ductile transition are contradicting, and we will weight the single contributions by performing carefully designed numerical experiments by means of two-dimensional discrete dislocation dynamics modelling. In our parameter studies, we vary the following parameters: (i) the mean free path for dislocation glide, *δ*, combined with (ii) the (obstacle) force of the grain boundary, *ϕ*, and (iii) the dislocation source spacing along the crack front, *λ*. Our results show that for materials or microstructures for which the mean distance of the intersection points of grain boundaries with the crack front is the relevant measure for *λ*, a decrease of grain size results in an increase of toughness. The positive impact of grain boundaries outweighs the negative consequences of dislocation blocking. Furthermore, our results explain the evolving anisotropy of toughness in cold-worked metals and give further insight into the question of why the grain-size-dependent fracture toughness passes through a minimum (and the brittle-to-ductile transition temperature passes through a maximum) at an intermediate grain size. Finally, a relation of the grain-size-dependence of fracture toughness in the form of *K*(*d*_*δ*_, *d*_*λ*_) = *K*_*IC*_ + *kd*_*δ*_^0.5^/*d*_*λ*_ is deduced.

## Introduction

The impact of grain size, *d*, on yield stress, *σ*_*y*_, can be described by the Hall-Petch relationship, as

1 $${\sigma }_{y}={\sigma }_{0}+{k}_{1}{d}^{-n}$$

where σ_0_ can be interpreted as the lattice friction stress, *k*_1_ is the Hall-Petch coefficient and the exponent *n* is approximately 0.5^1,2^. Equation (1) can be explained using pile-up models in which the Hall-Petch coefficient is a measure for the dislocation-grain-boundary interaction, i.e. the obstacle force, and where the grain size, *d*, can be regarded as the mean free path for dislocation glide, *δ*. While the experimental database for the Hall-Petch relationship displays a clear and unambiguous picture, the relationships between grain size and toughness, *K*, or grain size and the brittle-to-ductile transition (BDT) temperature, *T*_*BDT*_, are inconsistent.

Some experimental results suggest that a decrease in grain size results in an increase of toughness according to

2 $$K={K}_{0}+{k}_{2}{d}^{-n}$$

where *K*_0_ is toughness, *k*_2_ is a material constant and *n* is a positive exponent. Equation (2) is confirmed by the data reported by Curry and Knott^3^, Greenfield and Margolin^4^ and Srinivas *et al.*^5,6^. However, results of Werner *et al.*^7^ on the impact of grain size on toughness for α-brass and α-iron contradict Eq. (2). For the former material, he found that toughness decreases with a decreasing grain size, while for the latter Werner reported that toughness is not affected by grain boundaries^7^. Finally, Pacyna and Mazur^8^ found that toughness passes through a minimum at a specific grain size.

**Figure 1:**
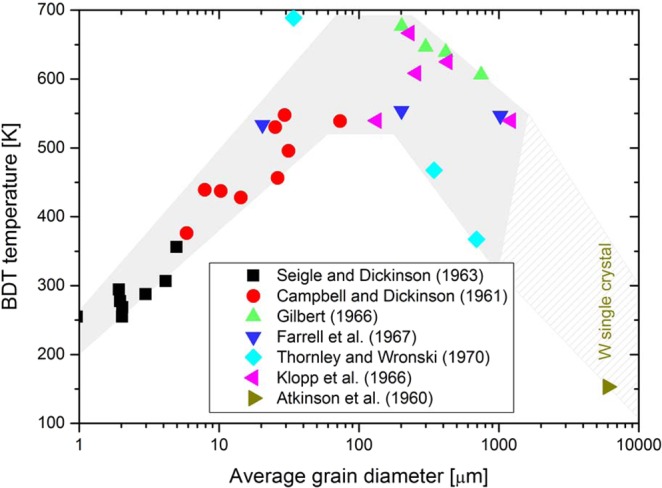
Experimental results of the effect of grain size on the BDT temperature of tungsten^21^. A similar behaviour has been reported by Tahmoush for iron (see Supplementary Figure 1)^22^. Data for single crystalline tungsten is from ref. ^23^.

As toughness is closely related with the BDT (macroscopically, the maximum in fracture toughness usually correlates with the transition from brittle to ductile material behaviour; thus the temperature at the maximum is taken as the BDT temperature), it comes as no surprise that the statements on the influence of grain size on the BDT temperature are controversial as well. Several investigators reported a decrease in the transition temperature with decreasing grain size^9–11^. For example, Bonnekoh *et al*. reported the results of a study in which several tungsten sheets have been rolled out from one sintered ingot^12^. The sheets differ in their degree of deformation, thus grain size, while the dislocation density was found to be constant. Bonnekoh *et al*. derived a relationship between the BDT temperature, *T*_*BDT*_, and the grain size, *d*, as

3 $${T}_{BDT}={T}_{0}-{k}_{3}{d}^{-n}$$

where *T*_0_ is a temperature, *k*_3_ is a material constant and *n* is a positive exponent. Bonnekoh *et al*. found the values *T*_0_ = 454 *K*, *k*_3_ = 72.3 K μm^0.5^ and $n=0.5$, where the grain size is given in microns^13^. In the Hall-Petch relationship, Eq. (1), the grain size, $d$, is interpreted as the mean free path for dislocation glide, $\delta$. However, in Eq. (3) the grain size, $d$, represents the mean distance of the intersection points of grain boundaries with the crack front, or more precisely, the distance of the dislocation source spacing, λ, along the crack front. Other investigators, for example Klopp and Witzke^14^, Gilbert^15^ and Thornley and Wronski^16^, have noted that the BDT temperature increases with a decreasing grain size. Both Farrell, Schaffhauser and Stiegler^17^ and Giannattasio and Roberts^18–20^ reported little or no dependence of the BDT temperature on grain size. In^18^ a comparison of single and polycrystalline material revealed no significant difference in the BDT temperatures or the apparent activation energy of the BDT. The latter suggest that this is an indication that dislocation motion near the crack tip is not significantly affected by the presence of grain boundaries. In a review paper, Stephens summarised the grain size dependence on the BDT temperature for tungsten^21^, by collecting data from refs. ^9,10,14–17^, and pointed out that interpretation of the data must be done with caution as the tested samples differ in their metallic purity. Nevertheless, the data suggest that the BDT temperature reaches a maximum at an intermediate grain size. For grain sizes less than 100 μm, the BDT temperature decreases with a decreasing grain size, while for grain sizes larger than 100 μm, the BDT temperature increases with a decreasing grain size (see Fig. 1)^21^. A similar behaviour has been reported by Tahmoush for iron (see Supplementary Fig. 1)^22^.

Clearly, the question of the ambivalent role of grain boundaries on toughness and the BDT has not yet been resolved. In simple terms, scholars are essentially split between two contradicting views, as summarized in Table 1. One group suggests that grain boundaries act as obstacles for gliding dislocations and that grain boundaries may confine the plastic zone size. Gliding dislocations are piled-up at grain boundaries and thus inhibit the instantaneous nucleation of further dislocations. Based on these considerations, toughness will decrease (and the transition temperature will increase) with a decreasing distance of the mean free path for dislocation glide, $\delta$, i.e. with decreasing grain size. The second group focuses on the intersection points of grain boundaries with the crack front and considers them as preferred sites for dislocation nucleation. This suggests that toughness increases (and the transition temperature decreases) with decreasing distance of the spacing of dislocation nucleation sites, λ, along the crack front, i.e. with decreasing grain size. Both mechanisms could be true for grain boundaries: (i) they are obstacles for gliding dislocations, but they are also (ii) preferred dislocation nucleation sites, particularly the intersection points of grain boundaries with the crack front. The question is just how to weight the contradicting contributions of each mechanism; which will be assessed within this paper.

**Table 1:** Summary of selected results on the influence of grain size on toughness and the BDT temperature. The results are contradicting; the issue remains unclear. The main objective of this paper is to harmonise the statements presented below, based on a dislocation dynamics model of crack-tip plasticity.

Topic	Statement	Material	Method	Authors
Toughness, K	Toughness **increases** with decreasing grain size, $d$	Mild steel; hot forged + heat treated; grain size range: 12–85 μm	K_IC_-testing; single edge notched bend (SENB) samples	Curry and Kott^3^
		α-β titanium alloy; heat treated to produce various grain sizes;	Charpy test geometry	Greenfield and Margolin^4^
		Armco iron; grain size range: 40–1050 μm	J_IC_-testing, single edge notched tensile (SENT) samples	Srinivas *et al.*^5,6^
	Toughness **decreases** with decreasing grain size, $d$	α-brass, cold rolled + recrystallised; grain size range: 10–175 μm	J-integral, compact tension samples	Werner^7^
	Toughness is **not affected** by grain boundaries	α-iron, 55% rolling reduction + recrystallisation; grain size range: 28–200 μm	J-integral, compact tension samples	Werner^7^
	Toughness passes through a **minimum** at a specific grain size	Tool steel; grain size range: 20–100 μm	K_IC_-testing, linear-elastic fracture mechanics	Pecyna and Mazur^8^
Britt-to-ductile transition	BDT temperature **decreases** with decreasing grain size, $d$	Recrystallised tungsten wires (wires made by powder metallurgy); grain size range: approx. 1–5 μm	Tensile testing; samples unnotched	Seigle and Dickinson^9^
		Recrystallised tungsten wires (wires made from melted tungsten); grain size range: approx. 10–50 μm	Tensile testing; BDT temperature was defined as the temperature which produces a 1% area reduction; samples unnotched	Campbell and Dickinson^10^
		Rolled tungsten plates; dislocation density = constant; grain size range: 0.37–1.1 μm (in the normal direction)	K_IC_-testing, linear-elastic fracture mechanics; single edge cracked tension (SECT) samples	Bonnekoh *et al.*^12,13^
	BDT temperature **increases** with a decreasing grain size, $d$	Tungsten, electron beam melted, swaged + recrystallised; grain size range: 200–1280 μm	Bend test; unnotched samples	Klopp and Witzke^14^
		Recrystallised tungsten; grain size range: 200–1000 μm	Bend test experiments; samples unnotched	Gilbert^15^
	BDT temperature is **not affected** by grain boundaries	Recrystallised tungsten; grain size range: 10–500 μm	Three-point bending tests; samples unnotched	Farrell, Schaffhauser and Stiegler^17^
		Tungsten single crystal; tungsten rod material, hot deformed; 3 μm mean grain size (cross section)	Four-point bend testing, pre-cracked samples	Giannattasio and Roberts^18–20^
	BDT temperature reaches a **maximum** at an intermediate grain size	Tungsten; grain size was achieved by annealing above the recrystallisation temperature	Summary of bending and tensile tests performed on unnotched samples	Stephens^21^
		Pure iron, cold rolling + recrystallisation; grain size range: 1–8000 grains per sq mm	Three-point bending test	Tahmoush *et al.*^22^

Against the background of these considerations, we identified the following three parameters as crucial for our assessment:

- the mean free path for dislocation glide, $\delta$
- the dislocation-grain-boundary interaction i.e. the obstacle force of the grain boundary, $\phi$
- the dislocation source spacing along the crack front, $\lambda$

In this paper we combine these parameters in well-defined case studies to answer the following main questions:

1. What is the role of grain boundaries (obstacles vs. sources) on toughness and the brittle-to-ductile transition? To what extent does the positive impact of grain boundaries (the intersection point of grain boundaries with the crack front are preferred sites for dislocation nucleation) outweigh the negative impact (grain boundaries are obstacles for gliding dislocations)?
2. Are we able to harmonise and to elucidate the contradicting experimental results by showing that toughness reaches a minimum at an intermediate grain size?
3. Can we provide explanations for the anisotropy of toughness in cold-deformed metals?
4. Will this study allow the refinement of our working hypothesis on the mechanisms controlling the BDT of pre-deformed, textured, polycrystalline body-centred cubic metals?

The paper is organised as follows: Background information on how we model crack tip plasticity is given in the next section. This is followed by the presentation and discussion of our results, which is subdivided into two main parts. In part one, we analyse the impact of grain size on toughness while in the second part we focus on the role of grain boundaries on the BDT. Finally, a brief conclusion is provided.

## Theory and Model

In this section, we describe essential features of crack tip plasticity and show how relevant properties are incorporated in a physical model describing crack tip plasticity by two-dimensional discrete dislocation dynamics. Finally, details on the computer experiments are given.

### Fundamental aspects of crack tip plasticity

**Figure 2:**
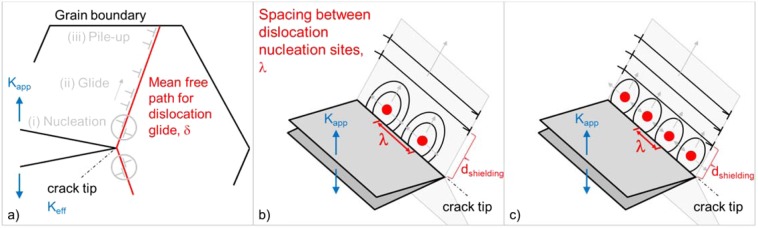
(**a**) Crack tip plasticity consists of the following subsequent processes: (i) the nucleation of dislocations, (ii) their glide through the crystal and (iii) their interaction with long-range obstacles such as grain boundaries. In this paper we assess the impact of the following three parameters on toughness and the BDT temperature: (i) the mean free path for dislocation glide, $\delta$, combined with (ii) the obstacle force of the grain boundary, $\phi$, both visualized in subfigure (**a**). In subfigures (**b**) and (**c**), the dislocation source spacing along the crack front, $\lambda$, and the process of nucleation of dislocation half-loops and their expansion and coalescence are demonstrated for a large source spacing (**b**) and a small one (**c**). After merging of the half-loops, the whole crack front is shielded. Dislocation sources along the crack front, can be discriminated into intrinsic sources, which operate within the grains, and extrinsic sources, as the intersection points of grain boundaries with the crack front. The dislocation source spacing, $\lambda$, impacts on the distance at which the half loops are merged, ${d}_{shielding}$.

The fracture toughness of semi-brittle materials is determined by the competition between bond-breaking at the crack tip and the mechanisms that govern crack tip plasticity^24^. The latter are shown in Fig. 2(a,b) and consist of the following sequential processes: (i) the nucleation of fresh dislocations, (ii) their glide through the crystal and (iii) their interaction with grain boundaries, i.e. pile-up formation^25^. Figure 2(a) discriminates between a global externally applied load, ${K}_{app}$, and a local, effective stress intensity factor, ${K}_{eff}$, at the crack tip. The effective stress intensity at the crack tip differs from the applied load, as the elastic fields from dislocations either increase (anti-shielding dislocations) or decrease (shielding dislocations) the effective stress intensity factor. The condition for onset of fracture is that the effective stress intensity reaches the critical stress intensity factor, ${K}_{IC}$, i.e. ${K}_{eff}=\,{K}_{IC}$. The nucleation of dislocations from dipole sources takes place in the vicinity of the crack tip. Dislocations that move towards the crack tip are anti-shielding dislocations. When they reach the free surface at the crack tip, they are annihilated and thus cause crack tip blunting. Dislocations that glide away from the crack tip are shielding dislocations. They form an inverse pile up combined with a dislocation free zone between the inverse pile up and the dislocation source^26^. A detailed image showing the features of crack tip plasticity can be found in ref. ^13^.

Figure 2 shows two relevant length scales that are crucial for crack tip plasticity and are in the focus of this paper: the mean free path for dislocation glide, $\delta$, and the spacing between dislocation nucleation sites, $\lambda$. Figure 2(b) shows two dislocation sources which operate in the vicinity of the crack tip and which emit dislocation half loops. Dislocation segments which glide parallel to the crack front direction are of the anti-shielding type. The half loops expand and merge. It is essential to recognise that the half loops must merge to provide effective shielding. The distance between the crack front and the position at which the half loops are merged is referred to as ${d}_{shielding}$ in Fig. 2(b,c) and is a function of the source spacing $\lambda$, thus

4 $${d}_{shielding}=f(\lambda )$$

which means that a decrease of the dislocation source spacing also decreases the distance at which the half loops are merged. Up to this merging, the crack front contains shielding and anti-shielding segments, while after the merging of the half loops the whole crack front is shielded. Furthermore, it is important to understand that a decrease of the dislocation source spacing, $\lambda$, does not result in an increase of the total amount of dislocations in the three-dimensional volume. Rather, the effect of a decrease of $\lambda$ is a decrease of ${d}_{shielding}$.

### Modelling crack tip plasticity in polycrystalline tungsten

We model crack tip plasticity by means of a two-dimensional discrete dislocation dynamic model. The model has been described in-depth in refs. ^27–30^. and is similar to that proposed by Roberts *et al*.^31–33^. In particular in ref. ^30^, the blocking of dislocations at grain boundaries and the influence of grain size on fracture toughness by this effect has been studied, however, neglecting the influence of grain size on dislocation nucleation and also disregarding temperature effects. Ingredients of the physical model are the Peach-Koehler force, the dislocation velocity, a dislocation nucleation criterion, periodic obstacles and crack tip blunting.

The Peach-Koehler force on the dislocations can be derived from the local stress field, ${\sigma }_{ij}$, which includes three contributions: the applied stress, ${\sigma }_{app}$, the image stresses due to the crack’s free surface, ${\sigma }_{image}$, and the stress fields of other dislocations, ${\sigma }_{dis}$. The Peach-Koehler force, ${f}_{PK}$, is related to the total resolved shear stress acting on the dislocations, $\tau$, as ${f}_{PK}=\tau b$, with the Burgers vector $b$. It can be transformed into a dislocation velocity according to

5 $$v={v}_{0}{(\frac{\tau }{{\tau }_{0}})}^{m}exp\{-\frac{Q}{{k}_{B}T}\}$$

where ${v}_{0}$ is a constant, ${\tau }_{0}$ is the unit stress, $m$ is the stress exponent, $Q$ is the activation energy and ${k}_{B}$ is the Boltzmann constant.

In this work, we consider crack tip plasticity as a symmetrical problem and thus apply two crack tip sources. The nucleation of the first dislocation occurs at ${K}_{eff}=0.2\,{K}_{IC}$, i.e. 20% of the critical stress intensity factor, ${K}_{IC}=\,\,$2 MPa m^0.5^. The subsequent dislocation emission will occur at progressively higher ${K}_{eff}$ values, which is due to the repulsive stress field from dislocations already emitted^31^. The two dislocation sources are located with an x-direction offset of 100 nm and a y-direction offset of 283 nm from the crack tip.

The freshly nucleated dislocations glide on slip planes including an angle $\Theta =\pm \,72.5^\circ$ to the crack plane. This angle corresponds to the highest resolved shear stress on the slip planes, as can be rationalised by considering closed-form expressions for the stress fields ahead of a crack tip for mode I in a linear elastic, isotropic material (^34,35^, see Appendix, A1).

Shielding dislocations glide away from the crack tip and pile up against grain boundaries, which are modelled as periodic obstacles with a given pinning force, $\phi$. Dislocations that glide towards the crack tip are anti-shielding dislocations, i.e. their stress field increases the local stress intensity at the crack tip. Furthermore, these dislocations increase crack tip blunting by their Burgers vector upon annihilation at the crack tip. The blunting of the crack tip is taken into account by increasing the critical local stress intensity factor as a function of the tip radius, ${r}_{tip}$. The radius-dependent value for the critical stress intensity, ${\hat{K}}_{IC}$, takes the form

6 $${\hat{K}}_{IC}({r}_{tip})={K}_{IC}\,(1+C\sqrt{\frac{{r}_{tip}}{{r}_{unit}}})$$

where C and ${K}_{IC}$ are constants. The constant ${r}_{unit}$ is the unit distance.

### Numerical experiments

The model presented above allows for the calculation of fracture toughness as a function of selected parameters, as temperature, loading rate and grain size. We varied the following parameters: (i) the mean free path for dislocation glide, $\delta$, combined with (ii) the dislocation-grain-boundary interaction, i.e. the obstacle force of the grain boundary, $\phi$, and (iii) the distance of the dislocation source spacing along the crack front, $\lambda$. These parameters are varied in four carefully designed studies, which will give new insight into the mechanisms controlling fracture toughness and the BDT. In case study I, the mean free path for dislocation glide, $\delta$, is varied, while the obstacle force, $\phi$, and dislocation source spacing along the crack front, $\lambda$, are held constant ($\phi$ = 1000 GPa; $\lambda$ = 0). In case study II we assessed the impact of dislocation source spacing along the crack front, $\lambda$, on toughness, while the other parameters remain constant ($\delta \,\nearrow \,\infty$, $\phi$ = 0). In case study III we combine case studies I and II and show how the simultaneous decrease of the dislocation source spacing along the crack front, $\lambda$, and the mean free path for dislocation glide, $\delta$, impact toughness. The latter is done by additionally varying the obstacle force, $\phi$. In addition, we perform a case study called “Minimum in toughness”. With this case study, we harmonise the contradictory statements presented in the introduction and explain why toughness passes through a minimum at an intermediate grain size and why the BDT temperature passes through a maximum at an intermediate grain size (see Fig. 1). Finally, we perform a case study to assess the evolving anisotropy in cold worked metals. In all our studies, the dislocation source spacing along the crack front, $\lambda$, and the force of the grain boundary, $\phi$, are assumed to be independent of the temperature. A summary of the selected parameters of the case studies is presented in Table 2.

**Table 2:** Overview of the three fundamental case studies performed and evaluated in this paper.

	Dislocation source spacing along the crack front, $\lambda$, [nm]	Mean free path for dislocation glide, $\delta$, [nm]	Force of the obstacle, $\phi$, [GPa]
**Case study I:** Variation of the mean free path, $\delta$	0	200, 400, 600, 800, 1000, 2000, 5000, 10000	1000
**Case study II:** Variation of the dislocation source spacing along the crack front, $\lambda$	200, 400, 600, 800, 1000	$\nearrow \,\infty$	0
**Case study III:** Simultaneous variation of the mean free path, $\delta$, and the source spacing, $\lambda$	200 (400, 600, 800, 1000)	200 (400, 600, 800, 1000)	0.1–1000
**Minimum in toughness:** Simultaneous variation of the mean free path, $\delta$, and the source spacing, $\lambda$, up to a critical value. Above this value, $\lambda$ is set constant and only $\delta$ is increased	${\lambda }_{c}^{1}$ = 400; ${\lambda }_{c}^{2}$ = 600; ${\lambda }_{c}^{3}$ = 800; ${\lambda }_{c}^{4}$ = 1000;	200–150000	0.3
**Anisotropy of the toughness:**	0	212, 318, 424, 530, 599, 1061, 1591, 2122, 2652, 2970	1000

**Figure 3:**
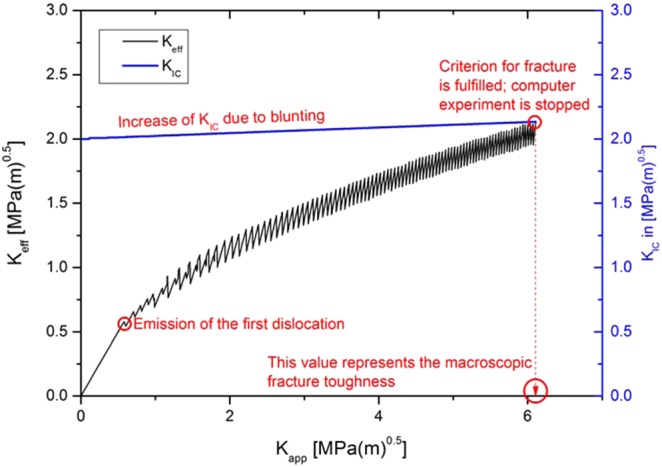
Effective stress intensity at the crack tip, ${K}_{eff}$, (black line, left axis) and critical stress intensity factor, ${K}_{IC}$, (blue line, right axis) plotted against the externally applied load, ${K}_{app}$. It is seen that each dislocation nucleation event causes a drop of the effective stress intensity, whereas the critical stress intensity increases with each absorption event. The simulation is stopped, when the effective stress intensity reaches the critical value, which defines the fracture toughness.

The simulations are conducted similarly to experimental tests, i.e. they start at zero applied load and without any pre-existing dislocations. The applied stress intensity is raised at a constant rate, $\dot{K}$ = 1 MPa m^0.5^/s. Pure tungsten is chosen as a model material and the used material properties are listed in Table 3. The numerical experiments are performed below the critical temperature, in the temperature range of 200–800 K with a temperature step of 200 K. The calculation is stopped as soon as the effective stress intensity at the crack tip, ${K}_{eff}$, reaches the radius-dependent value for the critical stress intensity, ${\hat{K}}_{IC}$ (${K}_{eff}=\,{\hat{K}}_{IC}$). The current global, externally applied load, ${K}_{app}$, is used as toughness, $K$, in the following figures. A plot ${K}_{eff}$ against ${K}_{app}$ is shown in Fig. 3.

**Table 3:** Material properties and model parameters for tungsten. More details, for example on the dislocation mobility law, can be found in ref. ^29^.

Parameter	Value
Shear modulus, $G$, [GPa]	152.67
Young’s modulus, $E$, [GPa] (plane strain)	393.88
Lattice constant, $A$, [pm]	315.9
Burgers vector, $b$, of *a*/2<111> screw dislocation [pm]	274
Poisson’s ratio, $\nu$	0.29
Angle between slip and crack plane, $\Theta$, [°]	70.5
Critical temperature [K]	810 K

## Results and Discussion

In this section, we present the numerical results of the parameter studies defined above. The parameter studies are designed to elucidate the impact of grain boundaries on toughness and the BDT. The studies will show to what extent the positive impact of grain boundaries (the intersection point of grain boundaries with the crack front are preferred sites for dislocation nucleation) outweigh the negative ones (grain boundaries are obstacles for gliding dislocations). Furthermore, the studies will evaluate whether it is possible to demonstrate that toughness reaches a minimum at an intermediate grain size. Finally, we defined parameter sets, which should give insight into the evolving anisotropy of the fracture toughness in worked metals.

In the following sections, we vary the parameters: (i) mean free path for dislocation glide, $\delta$, combined with the obstacle force of the grain boundary, $\phi$, and (ii) the distance of the dislocation source spacing along the crack front, $\lambda$. Among the latter, we distinguish between two types of sources: First, there are dislocation sources which operate at the crack front but inside the grains. These sources are well established from experiments on single crystals and are referred to as intrinsic sources. In this case, the relevant measure for $\lambda$ is the mean distance of the intrinsic sources. The dislocation source spacing of fractured single crystals can be made visible by etch pitting; images can be found in Riedle^36^. Second, we suggest that the intersection point of grain boundaries with the crack front are preferred dislocation nucleation sites. For materials or microstructures for which the mean distance of the intersection points of grain boundaries with the crack front is much smaller than the mean distance of the intrinsic sources, the relevant measure for $\lambda$ is the mean distance of the intersection points of grain boundaries with the crack front (Table 4).

**Table 4:** In this paper, we distinguish between two types of dislocation sources which operate along the crack front: First, there are intrinsic sources operating at the crack front but inside the grains. Second, we suggest the intersection points of grain boundaries with the crack front are preferred dislocation nucleation sites.

Comparison of the distances of the sources	Relevant measure for $\lambda$	Example, material/microstructure
mean distance of the intrinsic sources along the crack front << mean distance of the intersection points of grain boundaries with the crack front	$\lambda$ = the mean distance of the intrinsic sources	• Materials with narrow intrinsic source distances
		• Coarse-grained materials and single crystals
mean distance of the intersection points of grain boundaries with the crack front << mean distance of the intrinsic sources along the crack front	$\lambda$ = the mean distance of the intersection points of grain boundaries with the crack front	• Materials with broad intrinsic source distances
		• Fine-grained materials

With these considerations, we will now define and discuss the following case studies.

### Case study I: Variation of the mean free path (high obstacles forces)

In this case study, we assess the influence of grain boundaries on materials or microstructures that possess intrinsic sources that are very close to each other (relevant measure for $\lambda$ = the mean distance of the intrinsic sources). The mean free path for dislocation glide, $\delta$, is varied, while the force of the obstacle, $\phi$, and dislocation source spacing along the crack front, $\lambda$, are held constant ($\phi$ = 1000 GPa; $\lambda$ = 0). The force of the obstacle is set high, so that the grain boundary can be regarded as an impenetrable barrier.

**Figure 4:**
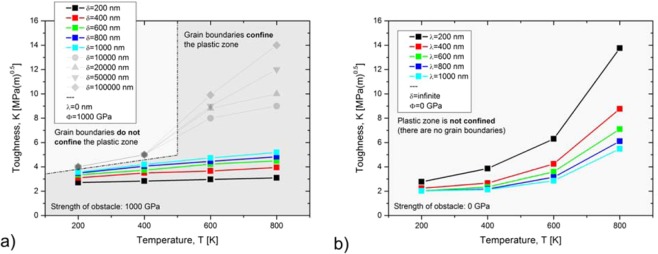
(**a**) Case study I ($\delta$ is varied, $\phi$ = 1000 GPa; $\lambda$ = 0): In case that grain boundaries confine the plastic zone, a decrease of the grain size (i.e. a decrease of the mean free path for dislocation glide, $\delta$) results in a decrease of toughness. (**b**) Case study II ($\delta$↗$\infty$, $\phi$ = 0; $\lambda$ = varied): A decrease of the dislocation source spacing, $\lambda$, results in an increase of toughness.

The results of the numerical experiments can be found in Fig. 4(a), where the fracture toughness, $\,K$, is plotted against the test temperature. Figure 4(a) distinguishes two regions highlighted in light and dark grey. The first one contains all data points for which the grain boundaries do not confine the plastic zone, meaning the dislocation that has been emitted first does not reach the grain boundary. The second one, highlighted in dark grey, contains all data points for which the grain boundaries confine the plastic zone. These data points result from numerical experiments in which the dislocation that was emitted first reaches the grain boundary and is pinned there.

In the light grey region, since the dislocations do not glide up to the grain boundary, the mean free path for dislocation glide is irrelevant. This explains why the data points are congruent at low temperatures. Furthermore, the toughness values slightly increase with increasing temperature, which can be traced back to the increase of dislocation velocity with temperature.

The data points in the dark grey box represent numerical experiments for which the grain boundary confines the plastic zone. After the emission of the first dislocation, it glides a certain distance until it is pinned at the grain boundary. Under such experimental conditions, Fig. 4(a) displays a clear and unambiguous picture of the role of grain boundaries on toughness: The smaller the grain size, the earlier the dislocations pile up, the more pronounced the suppression of fresh dislocations and the lower the toughness. Again, the slight increase of toughness with increasing test temperature can be explained by the temperature dependence of the dislocation velocity.

For materials or microstructures for which the relevant measure for $\lambda$ is the mean distance of intrinsic sources and for situations for which grain boundaries confine the plastic zone, a decrease of grain size (here: a decrease of the mean free path for dislocation glide, $\delta$) results always in a decrease of toughness. The experimental results on the impact of grain size on the toughness of α-brass by Werner can be explained by this mechanism^7^.

In this section, the impact of the mean free path for dislocation glide, $\delta$, on toughness was assessed. In the next section we will evaluate the impact of the dislocation source spacing, $\lambda$, on ${K}_{app}$.

### Case study II: Variation of the dislocation source spacing along the crack front

In this section, we present the results of case study II, in which the dislocation source spacing along the crack front, $\lambda$, is varied, while the mean free path for dislocation glide, $\delta$, and the force of the obstacle, $\phi$, are held constant. The case study is designed such that the mean free path for dislocation glide is set infinite, meaning the force of the obstacle is set to zero ($\delta$↗$\infty$, $\phi$ = 0). This setup guarantees that the plastic zone will never be confined and allows the investigation of the effect of $\lambda$ on ${K}_{app}$ in isolation.

The result of this case study can be found in Fig. 4(b), where the temperature-dependence of toughness is plotted for various values of $\lambda$. Consistently with the results from case study I, toughness increases with temperature. Moreover, a decrease of the dislocation source spacing, $\lambda$, results in an increase of toughness. This result was to be expected as a decrease of $\lambda$ results in a decrease of ${d}_{shielding}$ (see Fig. 2(b,c)).

Case studies I and II can be summarised as follows: if the mean free path for dislocation glide, $\delta$, is the only variable, a decrease of $\delta$ results in a decrease of toughness. However, if the dislocation source spacing along the crack front, $\lambda$, is the only variable, a decrease of $\lambda$ results in an increase of toughness. For a real microstructure we can then conclude the following: a decrease of grain size decreases the mean free path for dislocation glide which reduces the toughness (see case study I), but a decrease of grain size also decreases the dislocation source spacing along the crack front which results in an increase of toughness (see case study II). In the next section we will combine case studies I and II, i.e. we will simultaneously decrease the mean free path for dislocation glide, $\delta$, and the dislocation source spacing along the crack front, $\lambda$, to see whether one of these parameters outweighs the other and to compare the individual contributions of grain boundaries (sources vs. obstacles) on toughness.

### Case study III: Combined effect of mean free path and source spacing

In this case study, we assess the influence of grain boundaries on materials or microstructures for which the intersection points of grain boundaries with the crack front are the dominant dislocation sources (relevant measure for $\lambda$ is the mean distance of the intersection points). We assume that only the intersection points of the grain boundaries with the crack front are the dominant sources and that intrinsic sources (i.e. sources inside the grains) can be neglected. In case study III we combine case studies I and II and show how the simultaneous decrease of the dislocation source spacing along the crack front, $\lambda$, and the mean free path for dislocation glide, $\delta$, influence toughness. The latter is done by additionally varying the force of the obstacle.

**Figure 5:**
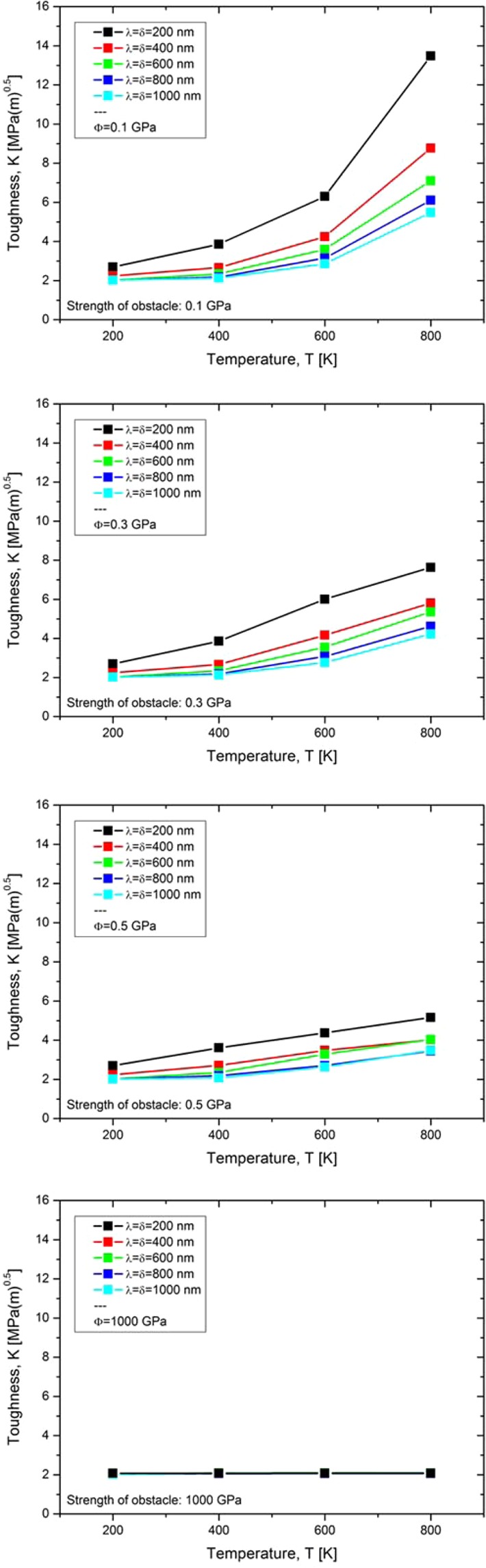
Case study III ($\delta$ and $\lambda$ are varied simultaneously, $\phi$ = 0.1, 0.3, 0.5 and 1000 GPa): Grain boundaries always have a positive impact on toughness. The positive impact of grain boundaries diminished with increasing strength of the obstacle, $\phi$.

The result of this case study can be found in Fig. 5, which shows a series of diagrams. The different diagrams result from studies that differ in the obstacle force. The force of the obstacle is varied from 0.1 via 0.3 and 0.5 up to 1000 GPa. The latter defines an impenetrable grain boundary. Assessing the positions of the dislocations at the end of the numerical test shows that for nearly all data points the first emitted dislocation reaches the obstacle. The data points at a test temperature of 200 K and a source spacing and mean free path for dislocation glide of 600, 800 and 1000 nm are the only exceptions. The results presented in Fig. 5 display an unexpected, but very clear picture: grain boundaries always have a positive impact on toughness. By the simultaneous decrease of $\lambda$ and $\delta$ from 1000 to 200 nm in 200 nm steps toughness increases. The positive impact of grain boundaries outweighs the negative one. This effect is more pronounced for low-strength obstacles and diminishes with increasing obstacle strength. However, there is never a situation where the negative impact of grain boundaries dominates fracture toughness. This result is of the utmost importance and is the fundamental basis for our attempt to transfer the numerical results to real materials and microstructures.

By comparing the results from case studies I and III we can conclude the following:

For materials or microstructures for which the relevant measure for $\lambda$ is the mean distance of intrinsic sources, and for situations for which grain boundaries confine the plastic zone, a decrease of grain size (here a decrease of the mean free path for dislocation glide, $\delta$) results in a decrease of toughness (Fig. 4(a)). However, for materials or microstructures for which the relevant measure for $\lambda$ is the mean distance of the intersection points of grain boundaries with the crack front, a decrease of the grain size (a simultaneous decrease of $\delta$ and $\lambda$) results in an increase of toughness. For such materials or microstructures, grain boundaries are always positive or neutral (Fig. 5). These results explain the experimental results on the impact of grain size on toughness from Curry and Kott^3^, Greenfield and Margolin^4^ and Srinivas *et al.*^5,6^.

We identified the situations in which a decrease of grain size results in a decrease or an increase in toughness. Based on this knowledge we are now able to set up a case study that demonstrates that toughness passes through a minimum at a specific grain size. This case study will be presented next.

### Fracture toughness passing through a minimum at a specific grain size

The work by Pacyna and Mazur demonstrates that toughness passes through a minimum at a specific grain size^8^. The aim of this section is to design a mechanism-based parameter setup that displays the grain-size-dependence of toughness and shows that toughness passes through a minimum at an intermediate grain size.

**Figure 6:**
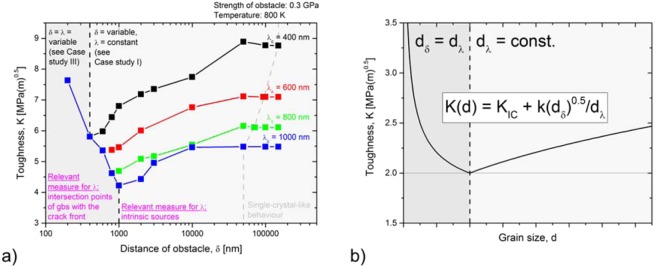
Toughness passes through a minimum at a specific grain size. (**a**) Results of the numerical experiments. The relevant measure for dislocation source spacing $\,\lambda$ changes at an intermediate, critical grain size. Above this critical value: $\lambda$ = const. (region highlighted in dark grey), below this value: $\lambda$ = $\delta$ = variable (region highlighted in light grey). (**b**) Analytical description.

The parameter setup used in this section is separated into two parts. Part one refers to the grain size region highlighted in light grey in Fig. 6(a) and describes coarse-grained material behaviour. For this region, the mean distance of the intrinsic sources along the crack front is much smaller than the mean distance of intersection points of grain boundaries with the crack front. In this case, the relevant measure for $\lambda$ is the mean distance of the intrinsic sources. For the parameter set representing the coarse-grained region, we choose $\lambda$ = const., $\phi$ = const., $\delta$ is variable (see case study I). Putting $\lambda$ as constant is reasonable, as the mean distance of the intrinsic sources along the crack front does not change with grain size. Part two of our parameter setup refers to the grain size region highlighted in dark grey in Fig. 6(a) and describes fine-grained material behaviour. For this region, the mean distance of the intersection points of grain boundaries with the crack front is much smaller than the mean distance of the intrinsic sources along the crack front. In this case, the relevant measure for $\lambda$ is the mean distance of the intersection points of grain boundaries with the crack front. For the parameter set representing the fine-grained region, we simultaneously modify $\lambda$ and $\delta$, with $\phi$ = const. (see case study III). Putting $\lambda$ as a variable is reasonable, as the mean distance of the intersection points of grain boundaries with the crack front changes with grain size. It is obvious, that the relevant measure for $\lambda$ changes with decreasing grain size from “the mean distance of the intrinsic sources along the crack front” to “the mean distance of intersection points of grain boundaries with the crack front”. It can be anticipated, that this transition takes place at an intermediate, critical grain size. The $\lambda$-value at this transition is referred to as ${\lambda }_{c}$ in Fig. 6(a).

Figure 6(a) displays the results of these assumptions. The toughness is plotted against the mean free path, $\delta$, on a logarithmic scale. The light grey region contains four graphs which are distinguished by their critical value for $\lambda$ (black: ${\lambda }_{c}$ = 400 nm; red: ${\lambda }_{c}$ = 600 nm; green: ${\lambda }_{c}$ = 800 nm; blue: ${\lambda }_{c}$ = 1000 nm). In this region, toughness decreases with decreasing grain size, consistent with case study I. The reason is the decrease of the mean free path for dislocation glide, caused by a decrease of the grain size at constant source spacing, which results in an early pile up and thus in an early suppression of the nucleation of further fresh dislocations. Note that the region highlighted in light grey contains a part labelled “Single-crystal-like behaviour”. Data points in this region result from numerical experiments in which the first dislocation which was emitted, travels a certain distance, but does not arrive at the grain boundary by the end of the test. The final positions of these dislocations with respect to the source spacing, $\lambda$, are summarised in Supplementary Table 1. At and below an intermediate, critical grain size, ${\lambda }_{c}$, we modified the parameter set. For the region highlighted in dark grey, we simultaneously modified $\lambda$ and $\delta$, because $\lambda$ is now dominated by the grain size $\delta$. The strength of the obstacle was held constant (*ϕ* = 0.3 GPa). In this region, a simultaneous decrease of $\lambda$ and $\delta$ results in an increase of toughness, consistent with case study III. Note that in the region highlighted in dark grey, the black, red and green curve are exactly below the blue curve and, thus, are not visible.

In Fig. 6(b), we finally arrive at an analytical expression for the toughness to grain-size relationship, $K=K(d)$. For this, we introduce the parameter $d$, representing the grain size. From this, it follows that ${d}_{\delta }$ is the mean free path for dislocation glide (= $\delta$), and ${d}_{\lambda }$ is the mean distance of dislocation sources along the crack front (= $\lambda$).

Therefore, we propose the relationship for the influence of grain size, $d$, on toughness, as

7 $$K({d}_{\delta },{d}_{\lambda })={K}_{0}+k\frac{{{d}_{\delta }}^{0.5}}{{d}_{\lambda }}.$$

For equiaxed grains and when the relevant measure for $\lambda$ is the mean distance of the intersection points of grain boundaries with the crack front, (i.e. ${d}_{\delta }$ = ${d}_{\lambda }$ = $d$) Eq. (7) simplifies to

8 $$K(d)={K}_{0}+k\frac{1}{{d}^{0.5}}.$$

This equation is exactly the equation presented in the introduction, Eq. (2), and describes the increase of toughness with decreasing grain size.

When the relevant measure for $\lambda$ is the mean distance of the intrinsic sources along the crack front, we consider ${d}_{\lambda }$ = $\lambda$ = constant (the temperature dependence of the intrinsic dislocation source spacing is neglected) and Eq. (7) can be written as

9 $$K({d}_{\delta })={K}_{0}+\frac{k}{{d}_{\lambda }}{{d}_{\delta }}^{0.5}.$$

In summary, we provided an explanation of the existence of a minimum of toughness at an intermediate grain size. The essential point is that relevant measure for $\lambda$ changes with decreasing grain size from “the mean distance of the intrinsic sources along the crack front” to “the mean distance of intersection points of grain boundaries with the crack front”. The point of this transition takes place at an intermediate, critical grain size, at which the fracture toughness assumes a minimum. In the domain above this critical grain size, we set $\lambda$ = const., and the toughness increases with grain size (see Fig. 6(a), region highlighted in light grey, or case study I, parameters: $\delta$ = variable, $\lambda$ = const.). In the domain below this critical grain size, we set $\lambda$ = $\delta$ = variable, and a decrease of grain size results in an increase of toughness (see Fig. 6(a), region highlighted in dark grey, or case study III, parameters: $\delta$ =$\,\lambda$ variable). With this, we can explain why toughness passes through a minimum at an intermediate grain size. Furthermore, we can harmonise the contradicting statements on the impact of grain size on toughness as presented in the Introduction.

Up to this point, we discussed the toughness properties of microstructures that possess equiaxed grains. In the next section, we will analyse the impact of elongated grains on toughness.

### On the anisotropy of the fracture toughness of worked products (rolled plates)

The aim of this section is to provide insights into the evolving anisotropy of toughness during cold working by the examples of cold rolled plates.

**Figure 7:**
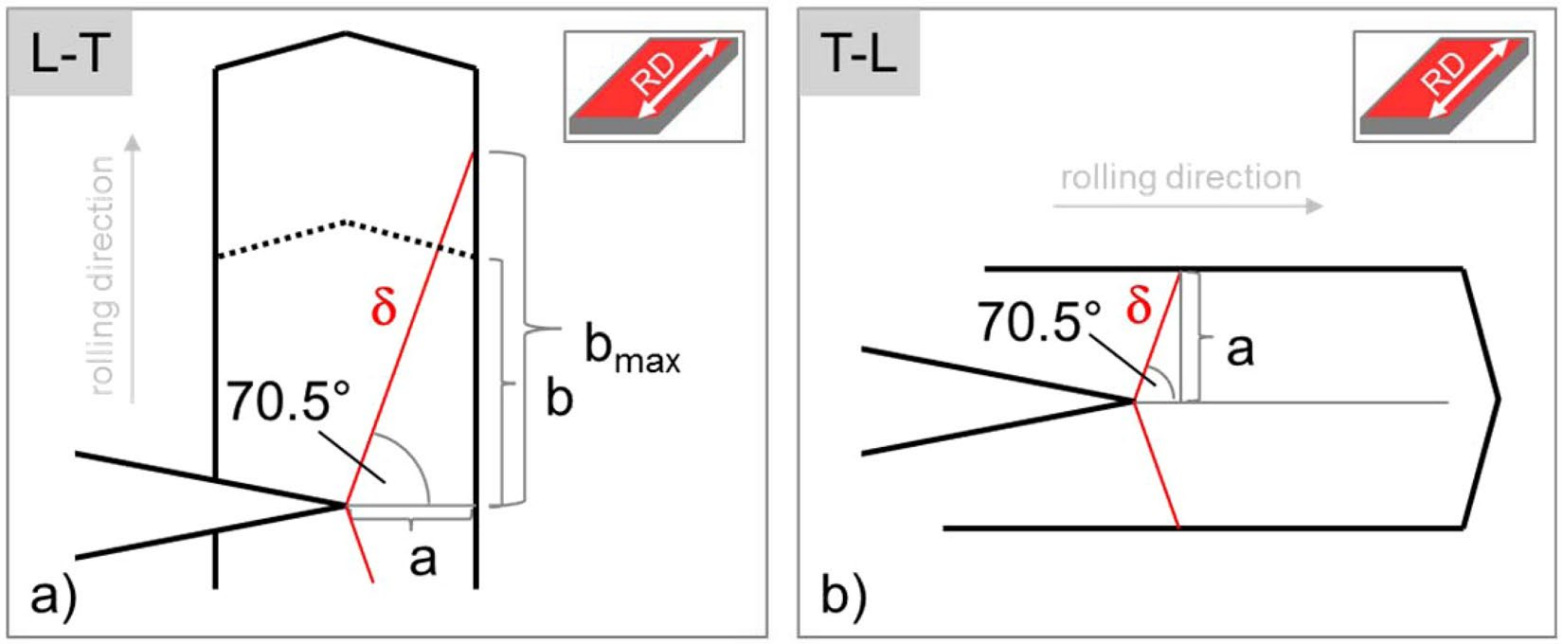
Schematic sketch of an L-T (**a**) and a T-L crack system (**b**). The images provide a view in the thickness direction of the rolled sheets (see the red surface of the insets). Based on geometrical considerations, a critical aspect ratio of tan(70.5) = 2.82 can be calculated (**a**).

In this section, we make use of the two-letter code introduced by the ASTM E399 standard^37^. The letter L stands for rolling direction, the letter T indicates the width direction and the letter S the thickness direction of the rolled sheet. Here, we define parameter setups to analyse the anisotropy of toughness of specimens aligned in the L-T and T-L reference directions. The first letter designates the direction normal to the crack plane, while the second letter gives the expected direction of crack propagation. For example, for the L-T specimen, the fracture plane normal is the L-direction (the rolling direction) and the expected direction of crack propagation is T-direction (the width direction). Figure 7 displays the above-mentioned crack systems and provides a view in the S-direction, the thickness direction of the sheet.

It is well established that toughness test samples representing an L-T crack system possess higher toughness values compared to samples representing a T-L crack system. Furthermore, experiments show that the BDT of L-T specimens occurs at a lower temperature compared to the transition temperature of samples representing a T-L crack system. Examples of experimental data on this issue can be found in the work by Reiser *et al.*^38^.

Rolling results in elongated grains. We consider such anisotropic grain shape by introducing the parameters $a$ and $b$. The former represents half of the chord length in the width direction and the latter stands for half of the chord length in the rolling direction. The parameters $a$ and $b$ define the grain size and the grain aspect ratio, respectively. It can be seen from Fig. 7(b), that parameter $a$ is directly linked with the mean free path for dislocation glide, $\delta$, according to

10 $$\delta =a/\sin (70.5^\circ ).$$

Figure 7(b) shows the relevant geometrical parameter of a T-L crack system. The relevant dimensions of an L-T crack system can be derived from a T-L crack system by rotating the grain from Fig. 7(b) by 90° around an axis parallel to the direction of the thickness of the plate. Figure 7(a) shows such an L-T crack system and contains a parameter $b$ which is linked to the mean free path for dislocation glide by a sinusoidal relation (see Eq. (10)). The mean free path for dislocation glide, $\delta$, increases with increasing value for $b$. However, this increase of $\delta$ is limited by a specific maximum value, which can be derived from a critical aspect ratio in the form

11 $${b}_{max}/a=\,\tan (70.5^\circ )=2.82.$$

A further increase of $b$, meaning a further increase of the aspect ratio, does not result in an additional increase of the mean free path for dislocation glide, $\delta$.

In the following parameter study, we vary the grain aspect ratio from 1 via 1.5, 2 and 2.5 up to the maximum value of 2.8. For half of the grain size in the direction of the width, we choose $a$ = 200 and 1000 nm. From this, together with the aspect ratios, values for $b$ and the mean free path for dislocation glide, $\delta$, can be calculated. The values are summarised in Table 5.

**Table 5:** Relation between parameter $a$, the grain aspect ratio and the mean free path for dislocation glide, $\delta$, with respect to the sample orientation (with respect to the crack system (L-T vs. T-L)). The different values for the mean free path for dislocation glide, $\delta$, cause the anisotropic fracture toughness in worked products such as rolled plates.

$a$	$b$	Grain aspect ratio [-]	L-T, distance of obstacle, $\delta$, [nm]	T-L, distance of obstacle, $\delta$, [nm]
200	200	1	212	212
	300	1.5	318	
	400	2	424	
	500	2.5	530	
	565	2.8	599	
1000	1000	1	1061	1061
	1500	1.5	1591	
	2000	2	2122	
	2500	2.5	2652	
	2800	2.8	2970	

For the study on the evolving anisotropy of toughness in cold rolled plates, we choose the following parameter setup: the mean free path for dislocation glide, $\delta$, is varied with respect to the grain size aspect ratio and the underlying geometrical relations for $a$ and $b$. Both, the obstacle force of the grain boundary ($\phi$ = 1000 GPa) and the distance of the dislocation sources along the crack front ($\lambda$ = 0) are held constant. Setting $\lambda$ constant can be rationalised by considering the microstructure along the crack front of samples representing a L-T and T-L crack system. The only parameter is the mean free path for dislocation glide, $\delta$.

**Figure 8:**
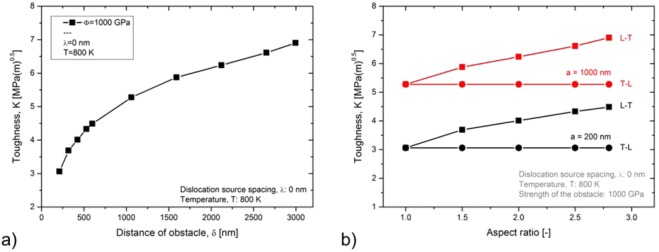
A decrease of the mean free path for dislocation glide, $\delta$, results in a decrease of toughness (**a**). From this and together with geometrical considerations the evolving anisotropy of toughness of rolled plates can be explained (**b**).

The result of this parameter study can be found in Fig. 8. In Fig. 8(a), the toughness is plotted against the distance of the obstacle or, in other words, the mean free path for dislocation glide, $\delta$. Toughness increases with increasing $\delta$. The same result was gained from case study I (see Fig. 4(a)). Furthermore, for all the data points plotted in Fig. 8(a), the grain boundaries confine the plastic zone; by the end of the numerical experiment, the first emitted dislocation is piled up at the grain boundary. The results from Fig. 8(a) can now be used to plot the anisotropy of fracture toughness. In Fig. 8(b), the toughness is plotted against the grain aspect ratio $a/b$. The data points in black display toughness values with a set to 200 nm, while data points in red display toughness values with a set to 1000 nm. An increase in the aspect ratio results in an increase of the toughness anisotropy, Fig. 8(b).

Rolling results in microstructures with anisotropic grain dimensions. The grain shape anisotropy together with the chosen crack systems (L-T or T-L) result in different values for the mean free path for dislocation glide, $\delta$ (Fig. 7). In agreement with the results from case study I, a decrease of the mean free path for dislocation glide, $\delta$, results in an early dislocation pile up at the grain boundary, an early suppression of the nucleation of further fresh dislocations from the source and, finally, a decrease in toughness. The experimental results on the anisotropy of toughness and the BDT temperature of rolled sheets by Reiser *et al*. can be reviewed^38^ against this background.

Up to now, the focus of the paper was to assess the impact of grain size on toughness. In the next section, we will arrive at a relationship between the grain size and the BDT temperature.

### From toughness data to the BDT temperature

The objectives of this section are as follows: First, we want to demonstrate that the numerical model can capture the transition from brittle to ductile material behaviour. Second, we want to introduce a pragmatic approach to determine the transition temperatures from our calculated toughness against temperature curves. And finally, we focus on the grain size dependence of the BDT temperature, ${T}_{BDT}=\,{T}_{BDT}(d)$. As already shown in Fig. 1, the ${T}_{BDT}$ to $\,d$ curve possesses a maximum at an intermediate grain size. An explanation for the shape of the curve will be given.

**Figure 9:**
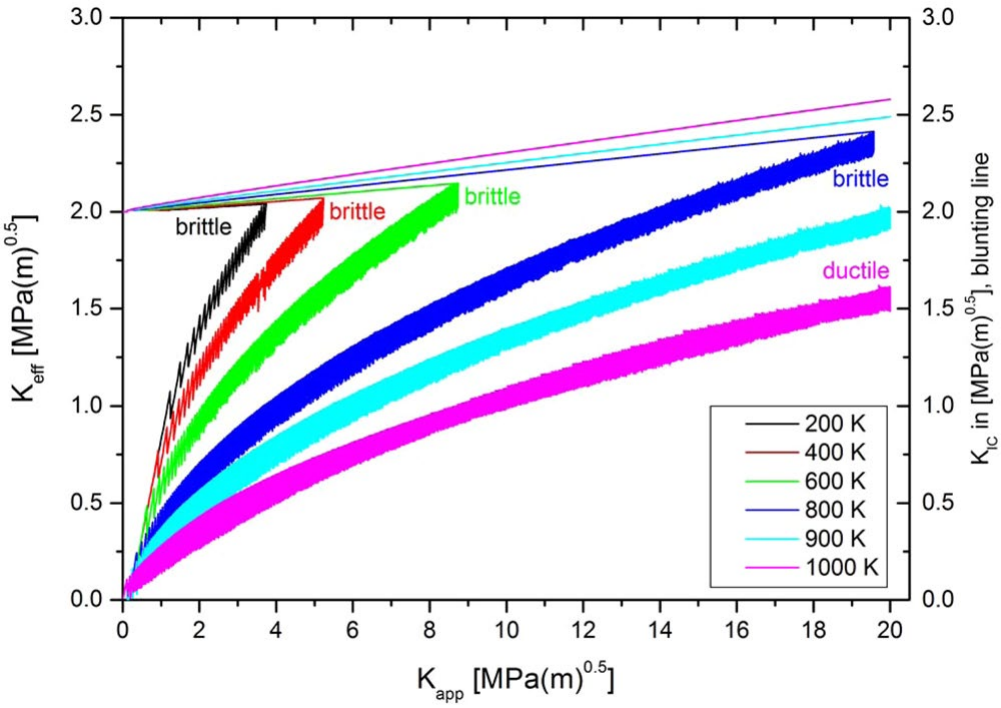
First definition of the BDT temperature. If ${K}_{eff}$ does not reach ${K}_{IC}$ for any ${K}_{app}$, the material behaviour is ductile, otherwise it is brittle. Furthermore, the slope of the curve representing the critical stress intensity, ${K}_{IC}$ to ${K}_{app}$, increases with increasing temperature. This clearly indicates that an increase in test temperature results in an increased blunting activity. Note: In Supplementary Figure 3, we plotted the number and position of the dislocations at the end of the experiments performed in this study.

In a first step, however, we need to assess a BDT temperature from our model. To accomplish this, we choose the most ductile parameter set, in which the dislocation source spacing is set to zero and no obstacles were used ($\lambda$ = $\phi$ = 0). The result of this study can be seen in Fig. 9, where the effective stress intensity at the crack tip, ${K}_{eff}$ (left y-axis), and the critical stress intensity, ${K}_{IC}$ (right y-axis) are plotted against the externally applied load, ${K}_{app}$. We define the material behaviour as brittle if ${K}_{eff}$ reaches the critical stress intensity, ${K}_{IC}$. Furthermore, we define the material behaviour as ductile, if ${K}_{eff}$ does not reach the critical stress intensity, ${K}_{IC}$, for any externally applied load, ${K}_{app}$. Figure 9 shows that for numerical experiments performed at 200, 400, 600 and 800 K, the effective stress intensity at the crack tip reaches the critical value. Thus, the material behaviour is termed as brittle. However, for the test performed at 1000 K the effective stress intensity does not reach the critical value even at an externally applied load of 20 MPa m^0.5^. Furthermore, the slope of the ${K}_{eff}$ to ${K}_{app}$ curve at 20 MPa m^0.5^ is very much like the slope of the curve representing the critical stress intensity, ${K}_{IC}$. From this it can be inferred that ${K}_{eff}$ does not reach ${K}_{IC}$ for any ${K}_{app}$. For such a situation, the material behaviour is termed ductile. Hence, our model is able to reveal a BDT temperature.

**Figure 10:**
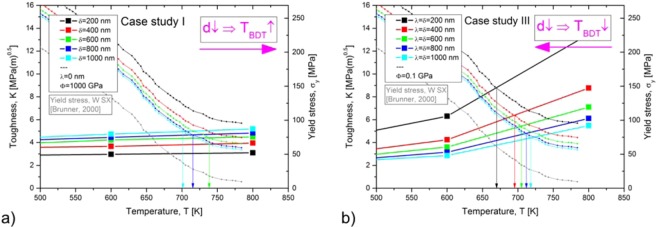
Second definition of the BDT temperature. The intersection of the toughness-over-temperature curve with the yield-stress-over-temperature curve gives the BDT temperature. (**a**) Variation of the free path according to case study I; (**b**) variation of source spacing together with free path according to case study III. The curves for the yield stress are obtained from experimental data that has been re-scaled according to the Hall-Petch relation.

Next, we want to introduce another definition of the BDT, which appears to be more pragmatic and which can be deduced directly from calculated toughness to temperature curves. Bonnekoh *et al.*^13^ showed experimentally that toughness values at and above the transition temperature scale with the temperature dependence of the yield stress, ${\sigma }_{y}$ (see Supplementary Figure 4). The scaling relation was found to be

12 $$K=A{\sigma }_{y}$$

where $A$ is a constant and has a value of 0.057. In Fig. 10, we used this value to scale the toughness (left y-axis) with the yield-stress (right y-axis).

Furthermore, Bonnekoh *et al*. ’s results show that curves representing the temperature dependence of toughness values in the brittle and semi-brittle regime intersect the ${\sigma }_{y}$ versus $T$ curves exactly at the transition temperature. From an experimental point of view, this intersection point is the point of a maximum in fracture toughness which usually correlates with the transition from brittle to ductile material behaviour; thus, the temperature at the maximum is taken as the BDT temperature. Therefore, we propose a second definition for the BDT: The intersection point of the calculated ${K}_{app}$ versus temperature curve with the ${\sigma }_{y}$ versus temperature curve gives the BDT temperature. The result of this procedure is shown in Fig. 10, where the toughness and the yield stress are plotted against the test temperature. Figure 10(a) uses toughness values as calculated in case study I, while Fig. 10(b) shows toughness data from case study III. The values for the yield stress are obtained by re-scaling experimental data for tungsten single crystals, taken from ref. ^39^, and plotted as grey curves. The re-scaled ${\sigma }_{y}$ versus temperature curves for polycrystals, were obtained from a parallel shift by making use of the Hall-Petch relationship, Eq. (1). Values for the Hall-Petch coefficient have recently been experimentally determined by Bonk *et al*.^40^. Here we used a value for ${k}_{1}$ of 15 N/mm^−1.5^ ^13^. For $d$ we used 200, 400, 600, 800 and 1000 nm respectively. In Fig. 10, we graphically determined the intersection points and marked the respective transition temperatures with arrows. It is noted here, that this procedure only yields semi-quantitative results for the BDT temperature, because the fracture toughness curves are obtained purely from or dislocation dynamics model, without fitting to fracture experiments, whereas the yield strength are obtained purely from experimental data, again without fitting to the model. Thus it is reassuring that the resulting BDT temperatures fall within a very reasonable range.

For case study I ($\delta$ is varied, $\phi$ = 1000 GPa, $\lambda$ = 0), a decrease in the grain size results in an increase of the BDT temperature. For case study III ($\delta$ = $\lambda$ simultaneously varied, $\phi$ = 0.1 GPa) a decrease in the grain size results in a decrease of the BDT temperature. An appropriate combination of both case studies should allow the construction of a ${T}_{BDT}$ versus $\,d$ curve which possesses a maximum at an intermediate grain size, which will be discussed next.

**Figure 11:**
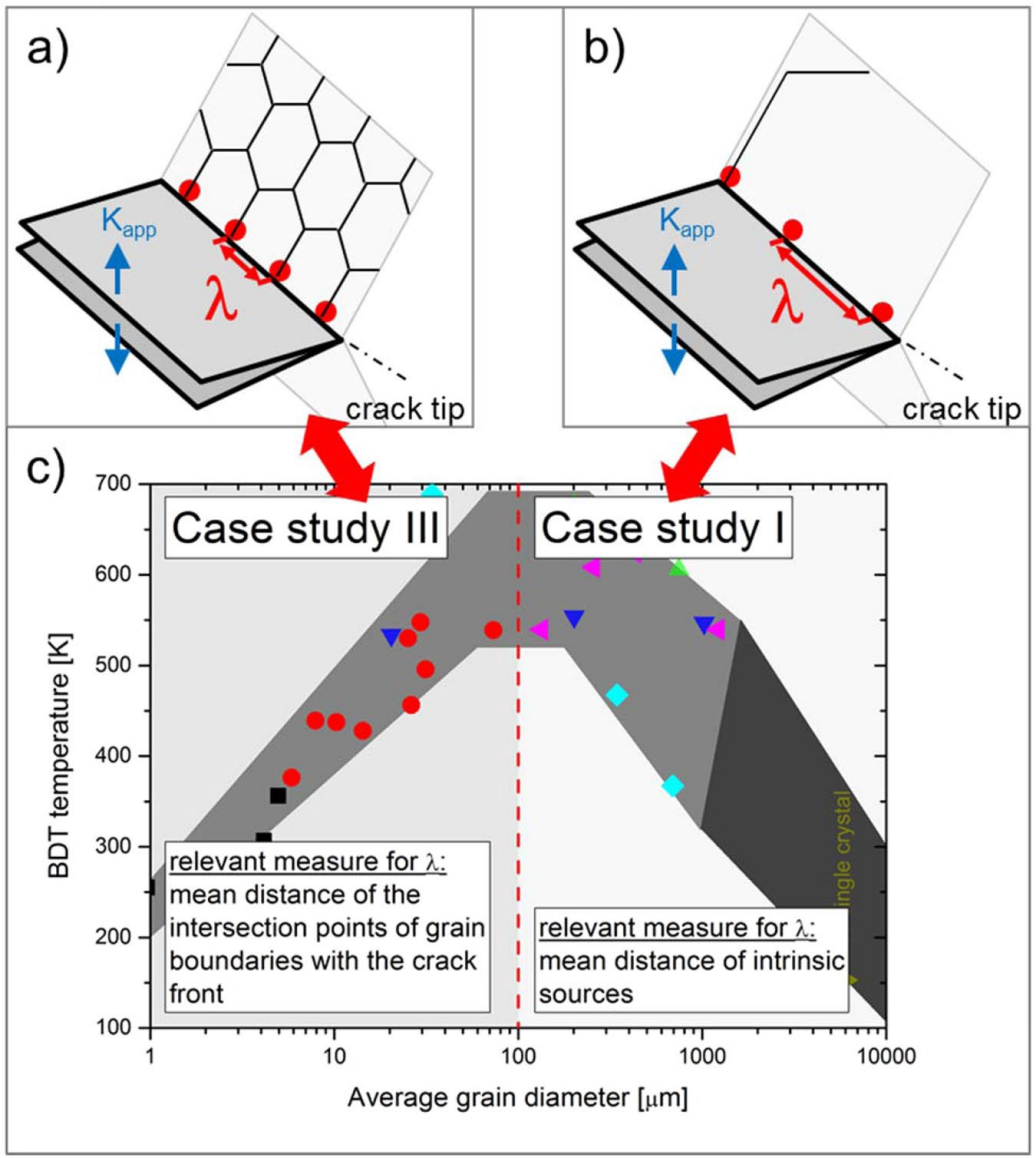
The BDT temperature passes through a maximum at an intermediate grain size. For materials or microstructures for which the intersection points of grain boundaries with the crack front is the relevant measure for $\lambda$, a decrease of grain size results in a decrease of the transition temperature (**a**). For microstructures for which the mean distance of intrinsic sources is the relevant measure for $\lambda$, a decrease of grain size results in an increase of the transition temperature (**b**). In (**c**), the data from Fig. 1 is discussed against the background of the mean findings of our numerical experiments.

In parallel with our explanation of the minimum of fracture toughness, we discuss the relevant measure for the dislocation source spacing, $\lambda$, first. It can be anticipated that the relevant measure for $\lambda$ changes with decreasing grain size from “the mean distance of the intrinsic sources along the crack front” to “the mean distance of intersection points of grain boundaries with the crack front”. For coarse-grained materials, the relevant measure for $\lambda$ is the mean distance of the intrinsic sources along the crack front (see inset (b) in Fig. 11). In this grain size regime, $\lambda$ can be treated as a constant; a decrease of grain size does not result in a decrease of $\lambda$. The results of this parameter setup can be seen in Fig. 10(a), which shows that the transition temperature increases with increasing grain size. At an intermediate, critical grain size the relevant measure for $\lambda$ changes. Below this critical grain size, the relevant measure for $\lambda$ is the mean distance of intersection points of grain boundaries with the crack front (see inset (a) in Fig. 11). In this grain size regime, $\lambda$ decreases with decreasing grain size. The results of this parameter set up can be seen in Fig. 10(b), which shows that the transition temperature decreases with a decreasing grain size.

Based on these results we arrive at the grain size dependence of the BDT temperature, as

13 $${T}_{BDT}({d}_{\delta },{d}_{\lambda })={T}_{BDT,0}-A\frac{{{d}_{\delta }}^{0.5}}{{d}_{\lambda }}$$

where ${T}_{BDT,0}$ and $A$ are constants. For equiaxed grains and when the relevant measure for $\lambda$ is the mean distance of the intersection points of grain boundaries with the crack front, (${d}_{\delta }$ = ${d}_{\lambda }$ = $d$) Eq. (13) simplifies to

14 $${T}_{BDT}(d)={T}_{BDT,0}-A\frac{1}{{d}^{0.5}}.$$

when the relevant measure for $\lambda$ is the mean distance of the intrinsic sources along the crack front, we can consider ${d}_{\lambda }$ = $\lambda$ = constant (the temperature dependence of the intrinsic dislocation source spacing is neglected) and Eq. (13) can be re-written as

15 $${T}_{BDT}({d}_{\delta })={T}_{BDT,0}-\frac{A}{{d}_{\lambda }}{{d}_{\delta }}^{0.5}.$$

## Conclusions

The aim of this paper was to identify the grain-size-dependence of toughness, $K=K(d)$, and the BDT temperature, ${T}_{BDT}=\,{T}_{BDT}(d)$, which was an open question before. On the one hand, grain boundaries are obstacles for dislocation glide. Gliding dislocations are piled-up at grain boundaries and thus inhibit the instantaneous nucleation of further dislocations at the crack. Based on these considerations, a decrease of grain size would result in a decrease of toughness. On the other hand, the intersection points of grain boundaries with the crack front are assumed to be preferred dislocation nucleation sites. A decrease of the dislocation source spacing along the crack front should then increase toughness. To weight the individual contributions of grain boundaries on toughness and the brittle-to-ductile transition, we performed carefully designed numerical experiments by means of two-dimensional discrete dislocation dynamics modelling.

In our parameter studies, we varied the following parameters: (i) the mean free path for dislocation glide, $\delta$, combined with (ii) the obstacle force of the grain boundary, $\phi$, and (iii) the dislocation source spacing along the crack front, $\lambda$.

In case study I, we varied the mean free path for dislocation glide, $\delta$, while the other parameters were held constant. The results show that a decrease of the mean free path for dislocation glide, $\delta$, (a decrease of grain size) results in a decrease of toughness. In case study II, we varied the dislocation source spacing along the crack front, $\lambda$, while the other parameters were held constant. The results show that a decrease of the dislocation source spacing along the crack front, $\lambda$, (a decrease of grain size) results in an increase of toughness. Case studies I and II show that the single contributions of grain boundaries (obstacles vs. source) on toughness are in fact contradicting. Therefore, we designed case study III to weight the single contributions. In this case study, we simultaneously varied the mean free path for dislocation glide, $\delta$, and the dislocation source spacing along the crack front, $\lambda$. The results show, that the simultaneous decrease of $\lambda$ and $\delta$ results in an increase of toughness. Under these test conditions, grain boundaries are always positive, or at least neutral.

The results of the fundamental case studies presented above, allow a mechanism-based explanation of why toughness passes through a minimum (and the BDT temperature passes through a maximum) at an intermediate grain size. To accomplish this, it is essential to see that the relevant measure for $\lambda$ changes with decreasing grain size from “the mean distance of the intrinsic sources along the crack front” to “the mean distance of intersection points of grain boundaries with the crack front”. This transition takes place at an intermediate, critical grain size. For materials or microstructures for which the relevant measure for $\lambda$ is the “the mean distance of the intrinsic sources along the crack front”, a decrease of grain size does not result in a decrease of $\lambda$, which can hence be regarded as a constant in this grain size regime. Under these conditions, a decrease of grain size results in a decrease of toughness (and an increase of the BDT temperature). However, for materials or microstructures for which the relevant measure for $\lambda$ is “the mean distance of intersection points of grain boundaries with the crack front”, a decrease of grain size results in a decrease of $\lambda$. Under these conditions, a decrease of grain size results in an increase of toughness (and a decrease of the BDT temperature).

The relationship between the grain size and toughness and the BDT temperature are in the form of equations (7) and (13).

## Supplementary information

Supplementary Material.

## Appendix

The shear stress component, ${\tau }_{r\Theta }$, of a stress fields ahead of a crack tip for Mode I in a linear elastic, isotropic material expressed in polar coordinates can be written as

A.1 $${\tau }_{r\Theta }=\,\frac{{K}_{I}}{\sqrt{2\pi r}}\,\sin (\varTheta /2)co{s}^{2}(\varTheta /2)$$

where ${K}_{I}$ is the toughness, $r$ is the distance from the crack tip and $\Theta$ is the angle between the crack and the slip plane. This trigonometric function reaches a maximum at $\Theta$ = 70.5° (see Supplementary Figure 2).
