# Supplementary material for: Elucidating the dual role of grain boundaries as dislocation sources and obstacles and its impact on toughness and brittle-to-ductile transition
Source: Sci Rep. 2020 Feb 17;10:2739. doi: 10.1038/s41598-020-59405-5 (PMC7026077; doi:10.1038/s41598-020-59405-5)
Supplement: Supplementary file 1 — Supplementary Material. [file 41598_2020_59405_MOESM1_ESM.pdf]

Elucidating the dual role of grain boundaries as dislocation sources and obstacles and its impact on toughness and brittle-to-ductile transition

Jens Reiser[a], Alexander Hartmaier[b,]*

a: Karlsruhe Institute of Technology, Institute for Applied Materials, 76344 Eggenstein-Leopoldshafen, Germany
b: Ruhr-Universität Bochum, Interdisciplinary Centre for Advanced Materials Simulation, 44780 Bochum, Germany

Corresponding author:
*: Prof. Dr. Alexander Hartmaier, ICAMS, Ruhr-Universität Bochum Universitätsstr. 150, 44801 Bochum, Germany, Phone: +49 234 32-29314, E-mail: Alexander.Hartmaier@rub.de

Supplementary Materials

Supplementary Figures

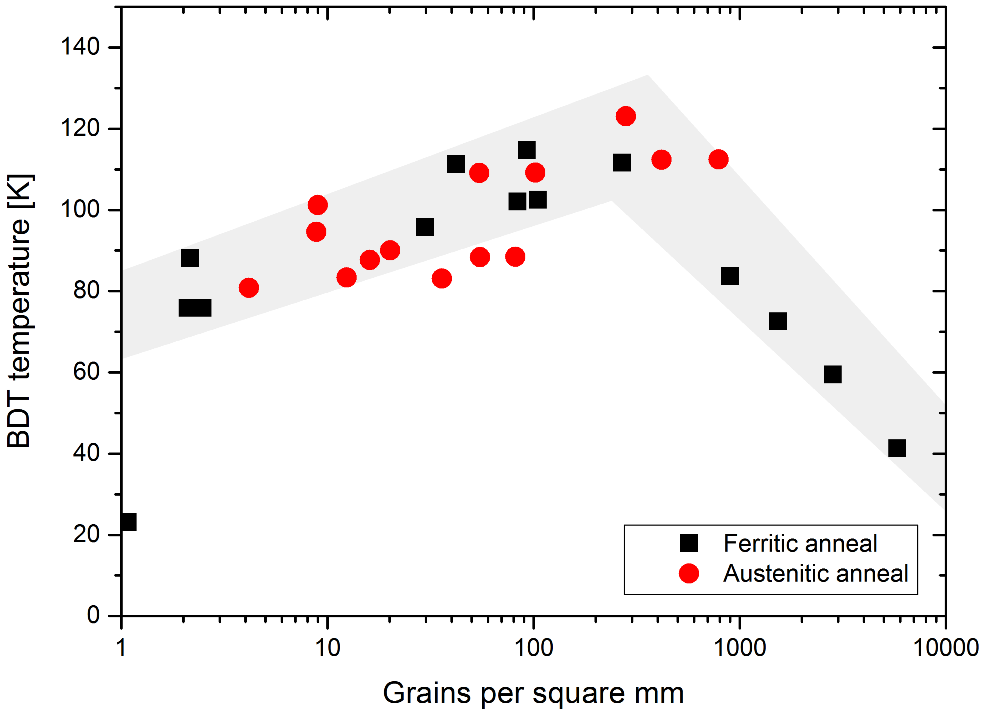

Supplementary Figure 1. The BDT temperature for pure iron reaches a maximum at an intermediate grain size. Data reproduced from Ref. [22].

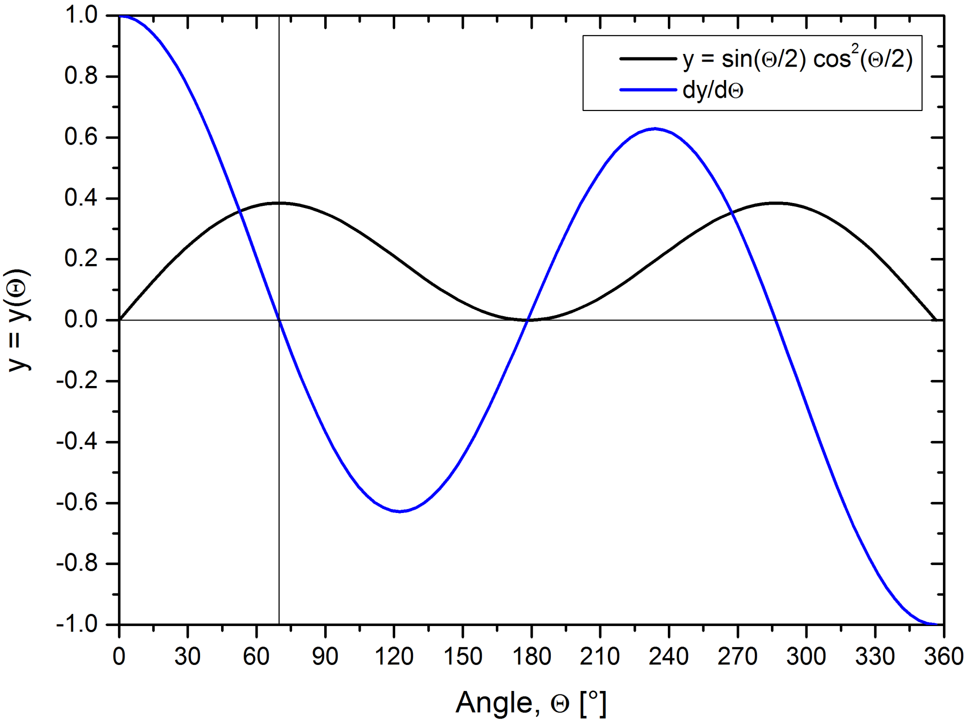

Supplementary Figure 2. The shear stress ahead of a crack tip reaches a maximum at Θ = 70.5° (see also Appendix A. 1).

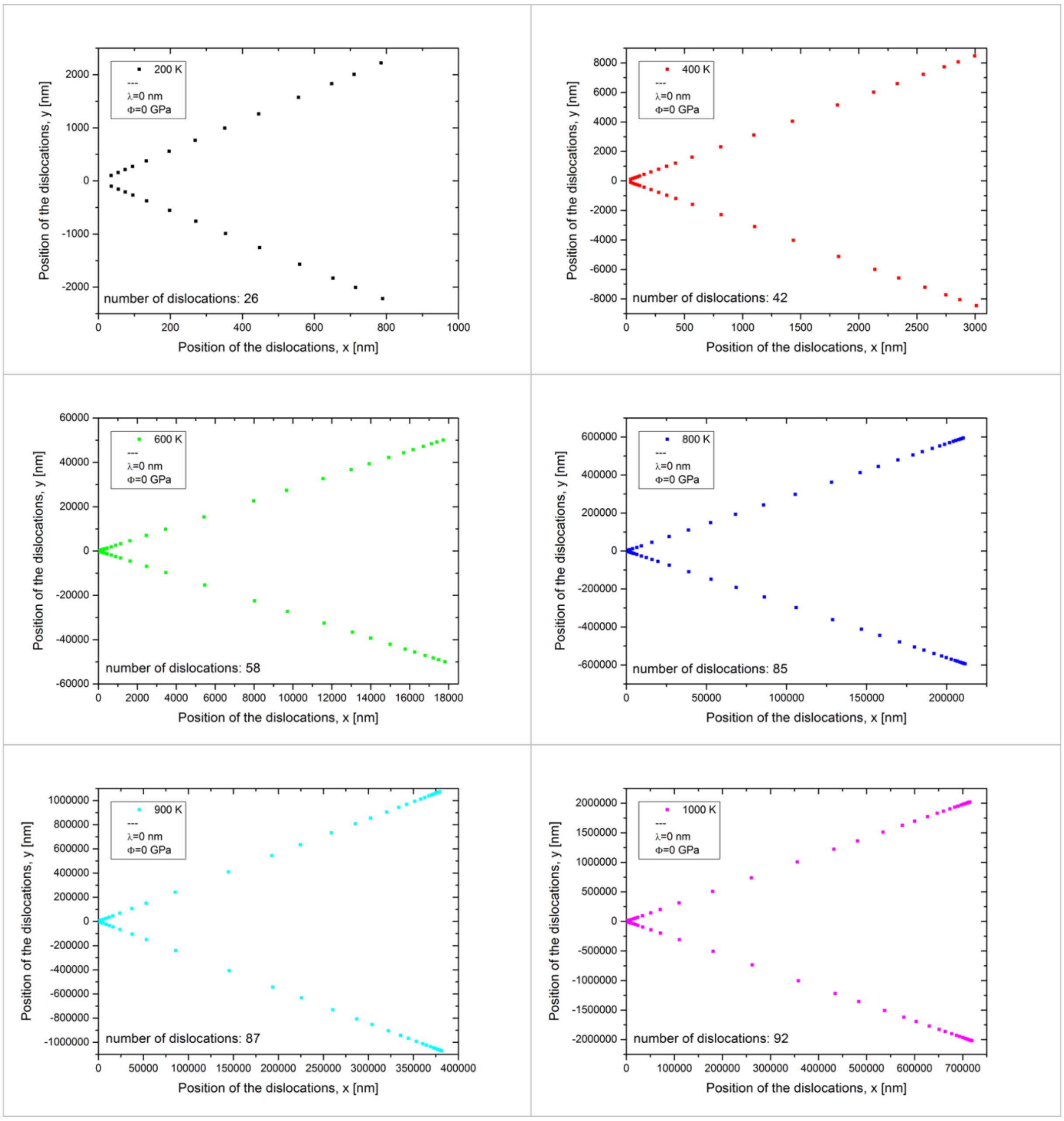

Supplementary Figure 3. Position of the dislocations at the end of the experiments. This figure is related to the experiments presented in Figure 9.

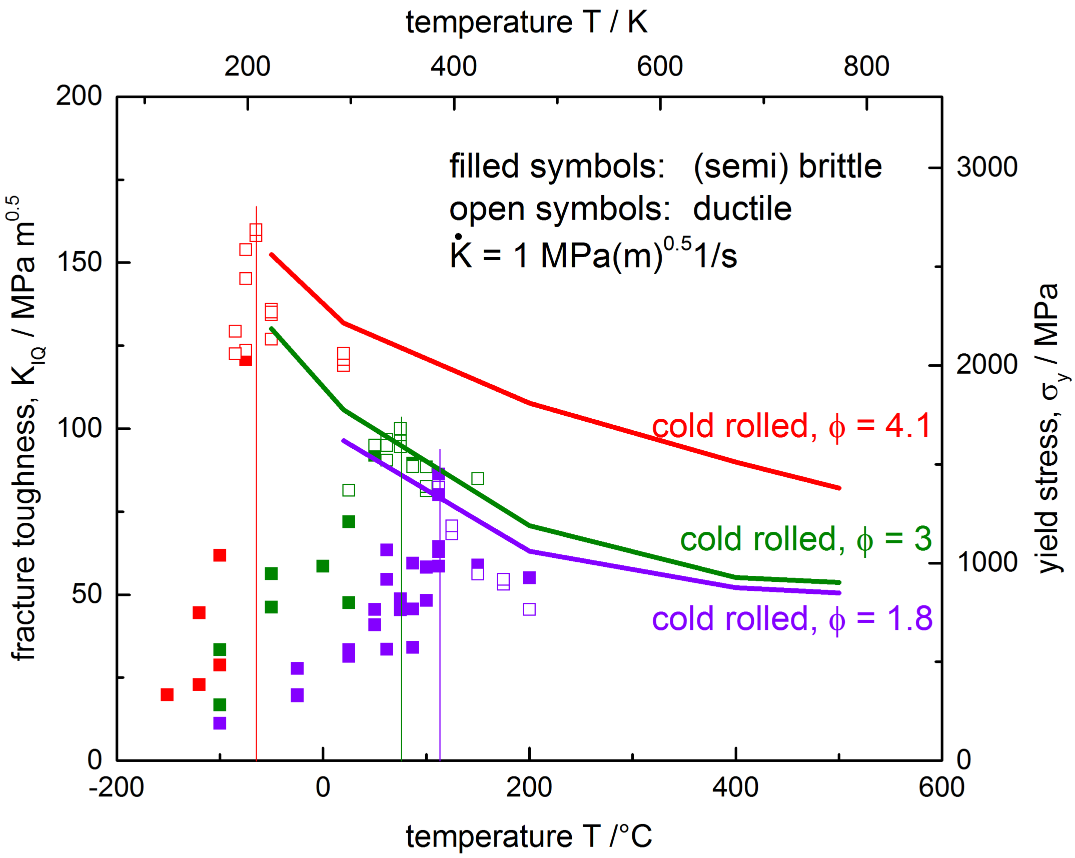

Supplementary Figure 4. Macroscopically, the maximum in fracture toughness correlates with the transition from brittle to ductile material behaviour. Experiments show that the maximum of the fracture toughness scales with the yield stress, σ_y. Figure from Ref. [13] (modified).

Supplementary Tables

Supplementary Table 1: Distance from the crack tip of the first emitted dislocation in a material with no obstacles. With respect to the results presented in Figure 6, we define the behaviour as single-crystal-like if the first dislocation does not reach the grain boundary.

Source spacing, λ, [nm]	Temperature [K]	Distance from the crack tip [nm]
400	800	139251
600	800	94620
800	800	71778
1000	800	58774
